# Evolution of molecular communication in the permanent *Azolla* symbiosis

**DOI:** 10.1111/nph.70699

**Published:** 2025-11-06

**Authors:** Deren Büyüktaş, Ellen Sigourney Lorberg, Sophie de Vries

**Affiliations:** ^1^ Evolution of Cyanobacterial‐Plant Symbioses Group, Department of Applied Bioinformatics, Institute of Microbiology and Genetics University of Göttingen Goldschmidtstr. 1 37077 Göttingen Germany; ^2^ Evolution of Cyanobacterial‐Plant Symbioses Group, Department of Applied Bioinformatics, Göttingen Center for Molecular Biosciences (GZMB) University of Göttingen Goldschmidtstr. 1 37077 Göttingen Germany

**Keywords:** molecular plant–microbe interaction, organelles, plant evolution, symbiotic cyanobacteria, symbiotic nitrogen fixation

## Abstract

Heritable symbioses exist across eukaryotes with different degrees of intimacy. In most cases, the symbionts are obligate and require inheritance for their survival. On the host side, symbiont retention can facilitate fitness benefits. Only rarely are these symbioses interwoven to the point that host survival relies on the symbiont. In land plants, the symbiosis of the water fern *Azolla* with its symbiotic cyanobacterium shows such a degree of high co‐dependence. The symbiosis originated in the last common ancestor of *Azolla* and exists continuously for at least 60 million years with no evolutionarily stable, secondary loss of the symbiont reported. This is a feat achieved by interactions on an organellar‐like level or those considered recent organelle acquisitions. Yet, *Azolla*'s symbiont is extracellular. How can loss of autonomy concomitant with full co‐dependence be accommodated in this extracellular symbiosis? Here, we synthesize what we know from the *Azolla* symbiosis on the consequences of evolutionary co‐dependence and stable symbiont retention. We discuss the need for symbiotic integration into environmental responsiveness if host survival depends on symbiont well‐being. Cross‐organismal integration of environmental stress responses may be one of the key steps that favor this evolutionarily stable permanent integration.

**Table:** 

	Contents	
	Summary	1666
I.	*Azolla* harbors a unique permanent symbiosis with a cyanobiont	1666
II.	Cyanobacteria in symbiosis: diversity and recurrent patterns	1668
III.	Balancing environmental input and symbiotic perpetuity	1670
IV.	Concluding remarks	1671
	Acknowledgements	1672
	References	1672

## I. *Azolla* harbors a unique permanent symbiosis with a cyanobiont

**Fig. 1:**
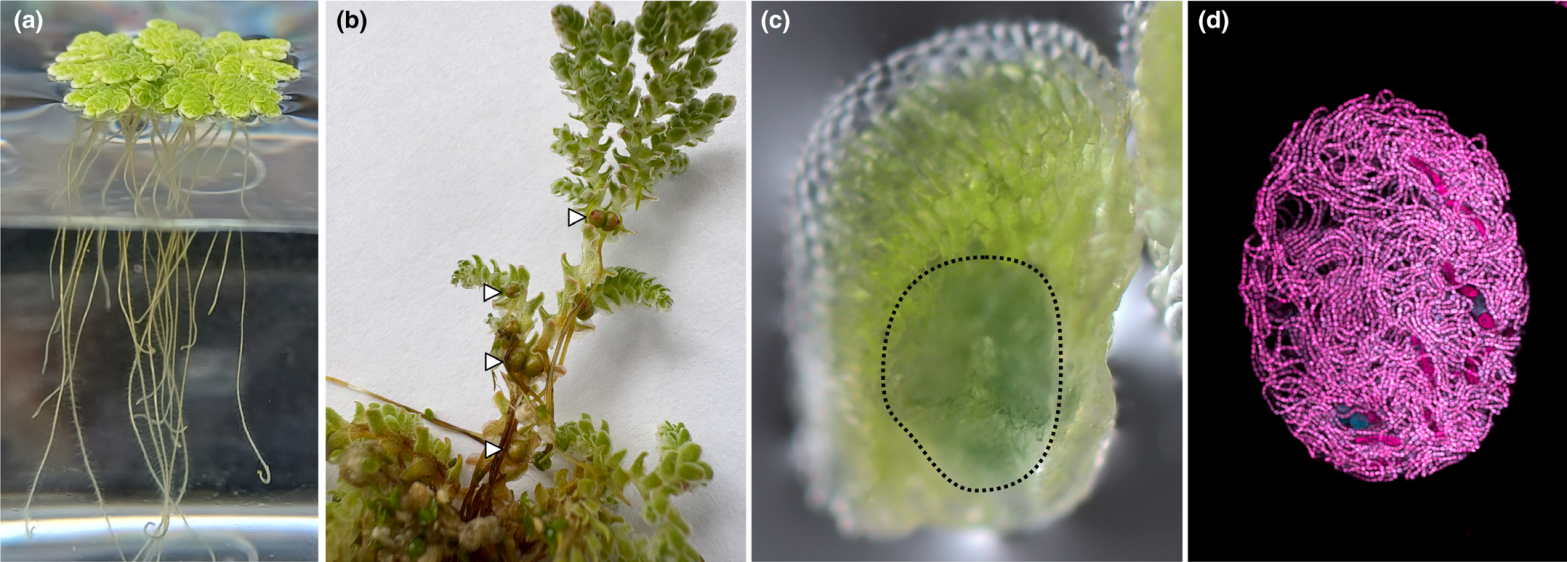
Water fern *Azolla* and its cyanobiont. (a) The fern *Azolla filiculoides* floating in a lab culture flask on International Rice Research Institute (IRRI) medium. (b) Close‐up of natural pond‐grown *Azolla* plants developing many microsporocarps that are marked with arrowheads. (c) A single leaflet picked from *A. filiculoides* grown in the laboratory; the leaf cavity filled with dark cyan‐green cyanobionts is clearly visible and outlined with a dotted line. (d) Confocal laser scanning micrograph of a leaf pocket isolated via digestion; cyanobiont autofluorescence was captured (623 nm emission).

The establishment of a permanent symbiosis is a complex process. It goes beyond mere inheritance of symbionts. It binds two organisms irreversibly together. Two such events led to the emergence of eukaryotes and Archaeplastida. Other than that, they remain rare. Yet, in land plants, the water fern *Azolla* harbors a vertically inherited nitrogen‐fixing cyanobacterial symbiont, a cyanobiont, in a special cavity below each leaf (e.g. Smith, 1955; Peters & Meeks, 1989; Fig. 1). Inheritance of symbionts exists in several plant lineages, such as grasses with *Neotyphodium* or the leaf nodule symbioses (Selosse & Schardl, 2007; Pinto‐Carbó *et al*., 2018). Neither has transitioned into permanency as host plants survive without symbionts, have symbiont‐free offspring (due to varying seed transmission frequencies), and/or re‐establish the symbiosis (Dahl Jensen & Roulund, 2004; Lemaire *et al*., 2012). Leaf nodule symbioses can persist without symbionts but eventually rely on re‐recruitment (Lemaire *et al*., 2012). By contrast, all species of the genus *Azolla* harbor the cyanobiont, established once > 60 million years ago (Ma) in the last common ancestor of *Azolla* (Ali *et al*., 2025). *Azolla* cannot live autonomously. With that, the *Azolla* symbiosis has transitioned into a permanent state. This is unique among land plants.

The twofold obligatory nature of the *Azolla* symbiosis leads to co‐dependence in fitness and survival of both partners: *Azolla* eventually dies in absence of a nitrogen source, when cyanobionts are lost (Brouwer *et al*., 2017; Güngör *et al*., 2024a). Artificial lab cultures of *Azolla*, where cyanobionts have been completely killed using antibiotics, show clear signs of stress and low resilience even upon nitrogen supplementation – without such supplements, they die. Cyanobionts appear incapable of autonomous growth (Gates *et al*., 1980; Gebhardt & Nierzwicki‐Bauer, 1991). Attempts to reintroduce (other) cyanobionts to the cavity of artificially cyanobiont‐free *Azolla* have failed (Watanabe, 1982). Indeed, leaf cavities from *Azolla* harbor only one strain of cyanobionts (Dijkhuizen *et al*., 2018; Armitage *et al*., 2025), although other cyanobacteria have been identified in surface‐sterilized *Azolla* (Song *et al*., 2025), suggesting that other cyanobacteria are not established in *Azolla*'s symbiotic structure. Other bacteria co‐inhabit the cavity and are potentially inherited (Zheng *et al*., 2009a; Dijkhuizen *et al*., 2018; Song *et al*., 2025), although this is not consistently observed (Armitage *et al*., 2025) and may depend on additional factors.

**Fig. 2:**
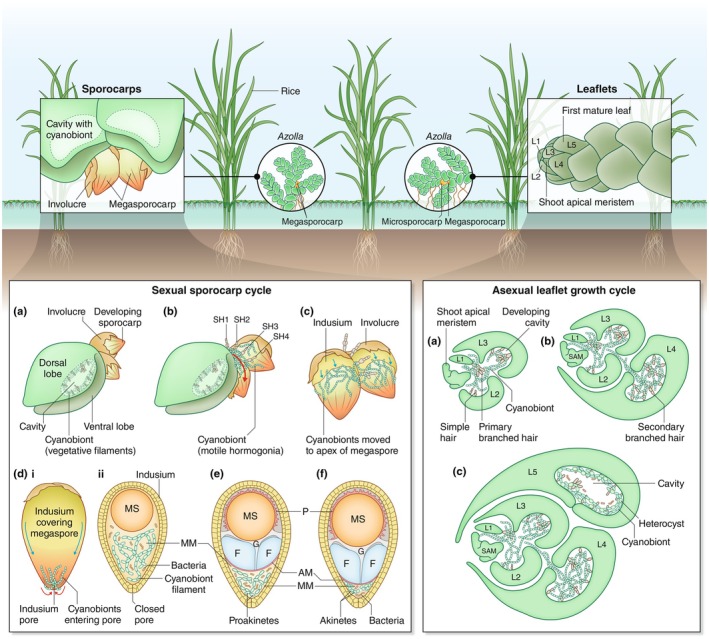
Transmission of cyanobionts in both reproductive cycles of *Azolla*. *Azolla* growing in a rice paddy. Insets show detailed views of megasporocarps and a shoot. *The sexual sporocarp cycle*. (a) Sporocarps grow in pairs (two microsporocarps, two megasporocarps, or a combination of both) at branching points (Dijkhuizen *et al*., 2021). Here, development of the megasporocarp is visualized. Initially, sporocarps are covered by an involucre which recedes as they mature. (c) Sporangial hairs (SH1–SH4) form at the base of the sporocarp pair, guiding cyanobionts from their motile, apical meristem colony to the sporocarps. (c) The indusium forms around the sporangial initial and begins to envelop it, mechanically separating the cyanobionts from the SHs. (di) Cyanobionts enter the megasporocarp through the indusium pore. (dii) Cross‐section of the megasporocarp showing the megaspore (MS) and the cyanobionts suspended in the mucilaginous matrix (MM). (e) The MS is covered by the perine (P). Between the cyanobiont population and the megaspore, a girdle (G), the floats (F) and an apical membrane (AM) develop. At the tip of the sporocarp, the indusium chamber containing the cyanobionts is located. During this developmental transition, cyanobionts differentiate into pro‐akinetes. (f) When the MS is mature, all cyanobionts are in an akinete stage. Once mature, the MS apparatus, containing MS, floats, and indusium chamber, will be released from the sporophyte by splitting the two‐layered megasporcarp and one‐layered megasporangial wall along the equatorial zone (not shown; Dunham & Fowler, 1987). The released MS apparatus appears to have an apical cap, also called the indusial cap, covering the floats and the indusium chamber, which is separated from the megasporangial wall, but still adheres to the float in the process of gametophyte maturation (Dunham & Fowler, 1987). When the first cotyledonary leaf penetrates the apical membrane, it engulfs cyanobionts in various stages (from akinetes to germinated cells) forming an inoculation chamber with four trichomes, the shoot apex and the first developing true leaves (Peters & Perkins, 1993). *The asexual leaflet growth cycle*. A schematic cross‐section visualizing leaf and cavity development of *Azolla*. Leaves are numbered (L1–L5) by their position relative to the shoot apical meristem (SAM). The SAM holds a motile cyanobiont population in hormogonia stage. (a) As the young leaves develop, the primary branched hair (PBH), a multicellular trichome, guides motile cyanobionts from the SAM to the developing cavity of L1. As cavities mature in L2–L3, simple, unbranched hairs form along the cavity. (b) The cavity encloses the primary branched hair, unbranched hairs and cyanobionts. A secondary branched hair forms within. Cyanobionts orient along the cavity sides and hairs and start to differentiate into vegetative filaments and form heterocysts. (c) By stage L5, the cavity pore is (nearly) closed. All cyanobionts have now differentiated into vegetative filaments with heterocysts. P, perine; Red arrows, movement of cyanobionts; blue arrows, growth of indusium layer.

How is permanent inheritance ensured? *Azolla* reproduces by vegetative growth followed by abscission of secondary rhizomes (Van Hove, 1989) or sexually through the generation of sporocarps (Smith, 1955; Fig. 2). During the growth of sporocarps, motile cyanobionts are guided to a pore where they enter the sporocarps (Peters & Perkins, 1993; Zheng *et al*., 2009b; Box 1; Fig. 2). Upon megaspore maturation, an apical membrane forms between the megaspore and the cyanobiont population, resulting in the formation of the indusium chamber (Peters & Perkins, 1993; Fig. 2). There, cyanobionts transition into akinetes (Grilli Caiola *et al*., 1992). During germination, the cotyledon leaf ruptures the apical membrane, giving the shoot apex access to the cyanobiont colony in the indusium chamber (Dunham & Fowler, 1987).

Box 1 **Glossary and background details**

**Abscisic acid:** One of the major phytohormones involved in the response to abiotic stress, whose signaling cascade is found across land plants and builds on an even older chassis (Cutler *et al*., 2010; Sun *et al*., 2019; Azar *et al*., 2025; Goldbecker & de Vries, 2025; Zimran *et al*., 2025).

**Akinetes:** Cyanobacterial developmental stage, which acts as a dormant, spore‐like state (Sukenik *et al*., 2019). In the cyanobiont of *Azolla*, the formation of these cells is coordinated with the life cycle of *Azolla* and under ambient conditions observed in the indusium chamber of the sporocarps.

**Cyanobiont:** Cyanobionts are cyanobacteria that live in obligate or facultative symbioses with other organisms. Cyanobionts are filamentous cyanobacteria belonging to the Nostocales. Facultative cyanobionts form two symbiotic clades (Warshan *et al*., 2018). The cyanobiont of *Azolla* has been named *Anabaena azollae*, *Nostoc azollae*, or lately *Trichormus azollae* (Pereira & Vasconcelos, 2014). A recent phylogenetic analysis placed it in a clade termed *Aphanizomenon* clade, within that forming a monophyletic clade with *Trichormus variabilis* PCC6309, *Anabaena cylindrica* PCC7122, *Sphaerospermopsis kisseleviana* NIES73, and *Raphidiopsis raciborskii* CS505, yet rapid radiation impacts phylogenetic inference across Nostocales (Pardo‐De la Hoz *et al*., 2023).

**Endosymbiotic gene transfer:** Transfer of genetic material from an endosymbiont to the genome of its host and functional integration of the endosymbiotic genetic material into the host genome.

**Heterocysts:** Heterocysts are the nitrogen‐fixing cells of filamentous cyanobacteria. They occur interspersed between vegetative cells and are characterized by a thicker cell wall. They ensure an oxygen‐poor environment for the oxygen‐sensitive nitrogenase. Unlike hormogonia formation, differentiation into heterocysts is an irreversible process.

**Hormogonia:** Hormogonia are the motile state of filamentous cyanobacteria. *Anthoceros*, *Gunnera*, and cycads secrete hormogonia inducing factors, HIFs, which induce hormogonia formation in facultative cyanobacteria. Gliding motility is facilitated by hormogonia‐specific polysaccharides (HPS), which are secreted near the cell membrane and function in tandem with the Type IV pili gliding motor (Gonzalez *et al*., 2019). The secretion of HPS is mediated by exosortases, for example CrtB identified in *Nostoc punctiforme* (Parrett *et al*., 2025).

**Jasmonic acid:** An oxylipin metabolism‐derived phytohormone that is known for its key roles in response to parasites and pathogens; the role of jasmonates in defense is found across land plants (Monte *et al*., 2018; Kneeshaw *et al*., 2022). Jasmonates can also act in diverse other plant processes (including development and reproduction; Stumpe *et al*., 2010).

**Lateral/horizontal gene transfer:** Transfer of genetic material between two different species and functional genomic integration of this material into the genome of the recipient species.

**Megasporocarp:** The megasporocarp is a structure built by the aquatic, heterosporous ferns that houses one female megaspore, which can be fertilized by male microspores. The megasporocarp is the smaller of both sporocarps in *Azolla*. The megasporocarps retain the cyanobiont population that will be transferred to the next generation.

**Microsporocarp:** The microsporocarps (Fig. 1b) house the microsporangia, which contain the microspores held together by foamy massulae. Upon release of the male spores, the cyanobionts are expelled to the environment and lost. The microsporocarp is the larger of the two sporocarp types of *Azolla*.

**Nitrogenase:** Nitrogenase is a multicomponent enzyme, which carries out the fixation of N_2_. It requires iron and molybdenum as co‐factors. Some nitrogenases accept vanadium as a co‐factor instead of molybdenum.

**Salicylic acid (SA):** A phenolic phytohormone with conserved occurrence across land plants and possibly in streptophyte algae (Jia *et al*., 2023; Kunz *et al*., 2024; Schmidt *et al*., 2024). SA is associated with the defense against abiotic and biotic challenges, with a potentially conserved antagonism to jasmonic acid‐based signaling (Matsui *et al*., 2020).

**Sexual inheritance in *Azolla*:** Sexual reproduction occurs naturally about once or twice a year (Watanabe, 1982). Its onset is associated with seasonal climatic changes, population density, and mat formation (Watanabe, 1982; Peters & Meeks, 1989) and inducible with far‐red light (Dijkhuizen *et al*., 2021). *Azolla* is heterosporous, producing two types of sporocarps, one containing male microspores, the other female megaspores (Peters & Meeks, 1989). Sporangial hairs guide cyanobionts to sporocarps before their sex is determined (Herd *et al*., 1985). Paternal cyanobionts are lost during the expulsion of microsporangia from the microsporocarps, leading to an exclusively maternal inheritance of cyanobionts (Peters & Meeks, 1989). Transmission of cyanobionts to sporocarps and cyanobiont development in sporocarps follow a coordinated process (Zheng *et al*., 2009a; Fig. 2): Sporocarps form at branching points (Dijkhuizen *et al*., 2021).

Every mature leaf of *Azolla* harbors the cyanobiont, ensuring that, upon vegetative propagation, every abscised plant carries the symbiont. Newly forming leaves receive part of the cyanobacterial population from the shoot apical meristem (SAM; Fig. 2). Each apical meristem contains a colony of motile cyanobionts, from which newly formed leaf cavities are inoculated via a trichome (Peters & Meeks, 1989). Retention of the apical colony may be one of the critical steps to ensure inheritance; leaf nodule symbioses with *Burkholderia* appear to also use an apical colony for inheritance (Pinto‐Carbó *et al*., 2018).

The consistent inheritance has left its evolutionary traces in the genomes of *Azolla* and cyanobiont. Patterns of co‐speciation are evident (Papaefthimiou *et al*., 2008; Li *et al*., 2018). The cyanobiont genome is eroded (Ran *et al*., 2010; Armitage *et al*., 2025), with more pseudo‐ and fewer protein‐coding genes compared with free‐living cyanobacteria (de Vries & de Vries, 2022). Carbohydrate metabolism, transport, and photosynthesis are under intensified selection (Armitage *et al*., 2025). The cyanobionts have higher heterocyst frequencies (*c*. 30%) than free‐living *Nostoc* (Meeks, 1998). The nature of this symbiosis has some organellar‐like characteristics, yet it lacks defining features of an organelle (Cavalier‐Smith & Lee, 1985; Keeling *et al*., 2015), such as endosymbiotic gene transfer (EGT; Li *et al*., 2018). This is likely due to the extracellular nature of the cyanobiont.

The *Azolla* symbiosis appears to be in an evolutionarily stable state of permanency – a state that in other associations may have been traversed before they became organelles. How is the coordinated co‐dependence of *Azolla* and its cyanobiont achieved in the absence of EGT and what are the necessary steps required to allow such evolutionarily continuous symbioses? In the following, we will set the *Azolla* symbiosis in context with recent discoveries of interactions with organellar character, as well as facultative cyanobacterial interactions.

## II. Cyanobacteria in symbiosis: diversity and recurrent patterns

Transitional stages between loose symbioses and becoming one organism remain scarcely explored. This process requires internalization, concomitant efficient communication, and host‐based control. Massive EGT has been suggested to be a major driver of increasing control and coordination between host and endosymbiont (de Vries & Gould, 2018).

The recently identified nitroplast found in marine algae has been coined an organelle‐like structure (Coale *et al*., 2024). Nitroplasts originate from nitrogen‐fixing cyanobacteria, are integrated within the host cell, and show host‐synchronized division. Owing to substantial genome reduction, they lost genes for autonomous metabolism – yet no traces of EGT are evident (Tripp *et al*., 2010; Suzuki *et al*., 2021; Coale *et al*., 2024). The related diazoplast of the diatom *Epithemia* likewise shows no evidence of EGT, although active DNA transfer occurs between symbiont and host nucleus (Frail *et al*., 2025). *Paulinella* and its chromatophore combine hallmarks, such as intracellularity, genome reduction, and a host–chromatophore synchronized life cycle, yet only a small number of genes derived from EGT (Nowack *et al*., 2011; Nowack *et al*., 2016; Gabr *et al*., 2020; Lhee *et al*., 2021). All associations appear relatively young with < 35 Ma (*Epithemia*‐diazoplast), *c*. 100 Ma (*Braarudosphaera bigelowii*‐nitroplast) or > 100 Ma (*Paulinella*‐chromatophore) and are in a similar range with the *Azolla* symbiosis (60–100 Ma). A significant accumulation of EGTs may require a longer evolutionary period. Thus, there need to be other means that can enhance coordination and control of two organisms on each other, particularly if both lose autonomy.

*Azolla* exemplifies that not even intracellularity is a prerequisite for permanency and tight coordination between life stages of symbiotic partners. *Azolla* and its cyanobiont must have established means of communication and compensation for their losses of autonomy. This requires intricate transport at trichomal contact points. Indeed, *Azolla* trichomes have cell wall ingrowths that in plants tend to be enriched with transporters (Zheng *et al*., 2009b). Ingrowths are enriched at cyanobiont contact sites (Zheng *et al*., 2009b). Yet, unlike, for example, the chloroplast, where evolutionarily dynamic recruitment of transporters to the chloroplast envelope may have replaced cyanobacterial transporters (Büyüktaş *et al*., 2025), the extracellular nature of the cyanobiont would suggest that cyanobiont transport systems were co‐opted for the symbiotic association. Consistently, Warshan *et al*. (2018) found an increased ‘carbohydrate transport and metabolism’ compared with facultative cyanobionts, while ‘inorganic ion transport and metabolism’ was reduced. Likewise, genes coding for transporters for the import of fixed nitrogen sources were reduced compared with free‐living cyanobacteria (Armitage *et al*., 2025). Yet, transporter remodeling is also observed for facultative cyanobionts, including transport of aliphatic and alkane sulfates, shown to be exchanged between symbiont and host (Stuart *et al*., 2020; Carrell *et al*., 2022), or secondary metabolites (Warshan *et al*., 2018). The predicted differently re‐shaped transporter sets may result from lifestyle‐dependent co‐option. It suggests that loose cyanobacterial interactions require different exchange than the permanent one of *Azolla*.

Different communication is logical given that both partners lost autonomy and are only able to live in association. One would expect a committed degree of exchange allowing one to sense and attune to each partner's developmental and physiological state. This is certainly also true for less‐committed symbioses, but they remain with the option of expelling symbionts upon, for example, a stressful physiological state (although it should be noted that beneficial symbionts can enhance the stress resilience of their host to some degree).

What do we know about other cyanobacterial–plant interactions regarding their environmental stress responses? Facultative cyanobionts exhibit distinct molecular and phenotypic traits enabling them to form associations with a wide range of plant hosts (Adams & Duggan, 2008; Rikkinen, 2017), for example, intracellular symbiosis with the angiosperm *Gunnera*, extracellular associations in mucilage‐filled cavities on thalli of liverworts and hornworts, epiphytic associations with mosses, and intercellular colonization of coralloid roots of cycads.

Success of cyanobacterial colonization depends on the environment. In hornworts, cyanobacterial associations are not determined by host genotype but rather soil community composition (Nelson *et al*., 2021). This is true for less and more intricate associations (Bouchard *et al*., 2020; Nelson *et al*., 2021). Furthermore, allelopathic interactions between symbiotic *Nostoc* were previously suggested (Liaimer *et al*., 2016), pointing to niche competition. Variation in N_2_‐fixing ability in successful cyanobionts may exist: the range of formed N_2_‐fixing heterocysts in symbiotic conditions is broad in most associations and variation in nitrogenase activity in symbiotic associations is reported (Meeks, 1998). *Azolla*'s retention of cyanobionts with varying degrees of N_2_‐fixing capability in similar environments is evident from reports of the former culture collection (Watanabe *et al*., 1992). Nevertheless, co‐evolution of 60–100 million years and the inability of re‐recruitment (Watanabe, 1982; Li *et al*., 2018) suggest that there is no retention of any potential punishing system in the *Azolla* symbioses. The evolutionary history leading to more or less efficient N_2_‐fixers and its interplay with environmental stressors remains to be further explored. Facultative associations with mosses suggest that N_2_‐fixation in cyanobionts depends on: (1) availability of essential co‐factors for nitrogenase as well as phosphate; and (2) external temperatures (Rousk, 2022). This hints that symbiotic efficiency seen as output of fixed nitrogen may depend on the environment. Whether cyanobionts are differentially recruited or present symbionts can be actively eliminated due to the physiological state of the host remains an open question. Yet, for noncyanobacterial interactions this is observed. Environmental stress, such as drought, leads to early senescence of root nodules, low nodulation, or decreased performance (Lie, 1971; Ruiz‐Lozano *et al*., 2001). Heat stress reduces the number of leaf nodules (Miller, 1990). Next, we will discuss what is known about how *Azolla* and its cyanobiont respond to environmental stress and set this in context with other intricate associations.

## III. Balancing environmental input and symbiotic perpetuity

In the cavity, the cyanobiont is surrounded by a diverse bacterial community, including denitrifiers potentially scavenging fixed nitrogen (Dijkhuizen *et al*., 2018). Some studies suggest that other bacteria are co‐inherited with *Azolla* (Zheng *et al*., 2009a; Song *et al*., 2025), yet: (1) others did not observe patterns indicative of co‐inheritance (Armitage *et al*., 2025); and (2) whether potential co‐inherited bacteria include putative cheaters remains unknown. Nonetheless, all investigated cavity microbiomes show changing diversity, suggesting that the cyanobiont has to remain responsive and resilient to other microbial organisms in the leaf pocket. Next to potential cheaters and other microbes that co‐occur with the cyanobiont, *Azolla* can be readily colonized by pathogens (Lumpkin & Bartholomew, 1986). Immune responses must be efficient against those pathogens but not the cyanobiont.

First explorations into the immune response of *Azolla* exist. Biotic cues such as cornicinine, a metabolite produced by crane flies, which co‐habit ditches in which *Azolla* dwells, significantly impact *Azolla* health (Güngör *et al*., 2024b). The triketide‐δ‐lactone causes chlorosis specifically on *Azolla* leaves, triggers akinete differentiation of the cyanobiont, and inhibits akinete transition into vegetative cells upon spore germination (Güngör *et al*., 2024b). Thus, biotic cues can significantly affect both symbiotic partners to a degree that both will die when failing to adjust to environmental stress.

In the above example, both cyanobiont‐free and akinete‐harboring cavity appear to induce a jasmonic acid (JA)‐governed defense pathway (Güngör *et al*., 2024b). Yet, methyl JA (MeJA) has no gross effect on cyanobacterial abundance (de Vries *et al.*, 2018); JA's influence on cyanobacterial development remains to be investigated. *Azolla* has multiple homologs to the JA receptor COI1 due to a fern‐wide COI1‐family expansion (Ali *et al*., 2025). Given the shift in preference from the jasmonate *dn*‐OPDA in bryophytes to JA‐Ile in angiosperms (Monte *et al*., 2018), it is challenging to infer jasmonate affinity of fern COI1 homologs.

The external application of another defense‐associated phytohormone (Box 1), salicylic acid (SA), leads to increased cyanobacterial abundance (de Vries *et al.*, 2018). SA levels are changeable in an artificially cyanobiont‐free culture in a nitrogen‐dependent manner in *Azolla,* and genes in SA signaling were responsive to the absence of the cyanobiont (de Vries *et al*., 2023). This points to a positive role and potential feedback loop for SA in this symbiosis. It is conceivable that this defense response has been co‐opted to be coordinated with the permanent cyanobiont. By contrast, grass hosts deficient in *Neotyphodium occultans* have lower SA levels compared with symbiotic ones, and higher levels of SA lead to decreased herbivore resistance conferred by the presence of the endophyte (Bastías *et al*., 2018). This suggests a trade‐off rather than co‐option for the *Neotyphodium*‐grass associations.

Continuous cold stress was found to trigger red coloration of cyanobionts, followed by disintegration of their filaments and eventual disappearance from the cavities (Güngör *et al*., 2024a). Concomitantly, the same study showed that *Azolla* plants produce red deoxyanthocyanin pigments that accumulate in proximity to and around the cavity. Only *Azolla* species with sufficient nutrient supply were able to recover, coherent with nutrient deficiency having similar effects and leading to the death of the cyanobionts before the death of *Azolla* (Watanabe, 1982). One species, though, recovers independent of environmental factors. Upon recovery, leaf cavities with cyanobionts emerged in newly formed leaves, suggesting a cold‐tolerant colony at the SAM. Noteworthily, in the presence of the phytohormone abscisic acid (ABA) some *Azolla* species recovered better, and ABA delayed red pigmentation and withering (Güngör *et al*., 2024a). This suggests tight ABA‐ and flavonoid‐based signaling loops with cyanobiont signaling, fitting past reports on flavonoids impacting cyanobacterial development (Cohen & Yamasaki, 2000; Cohen *et al*., 2002).

Together, these first insights into the stress response of the *Azolla* symbiosis suggest that: (1) stressful environments affect cyanobiont biology while *Azolla* adjusts cavity signaling; and (2) if cyanobionts are not rescuable, *Azolla* dies. Furthermore, some signals that accumulate during stress positively modulate cyanobiont development and/or abundance. In the case of SA, the cyanobiont appears to have gained some control over the stress response. Whether this is the case for other stress signaling responses remains to be investigated. Yet, from other eukaryotic associations we know that such coordination is evident. Pea aphid populations with *Buchnera* symbionts, where symbionts harbor a single‐nucleotide deletion in the promoter of a heat‐shock transcription factor, show higher reproductive rates under cool temperatures compared with aphid populations with *Buchnera* that have no deletion in that promoter (Dunbar *et al*., 2007). Moreover, in plants the chloroplast constitutes a conserved stress hub, showcasing the integration of stress signaling of two organisms.

Beneficial symbionts confer stress resilience to their host up to a certain point. But strong environmental stress can negatively impact symbiotic interactions. The more intricate the symbioses the more the symbiont depends on its host's well‐being – and eventually the host on that of the symbiont. In the *Azolla* symbiosis, it has passed the point where loss of symbiont means loss of benefits and reduced fitness, but translates into death. In this permanent symbiosis, we see first signs of an integrated stress response fine‐tuned between both partners. We thus hypothesize that an integration of stress response into symbiotic communication is a prerequisite, which allows for permanent dependence on the cyanobiont.

## IV. Concluding remarks

The establishment of cyanobiont‐deficient lines and subsequent molecular investigations have provided first insights into communication between *Azolla* and its cyanobiont. To unravel the evolutionary forces that drove this symbiotic integration to the point of inseparability, we need to clarify how their two genomes communicate. Key outstanding questions include: what is exchanged between host and symbiont beyond nutrients? At what developmental stages do these exchanges occur? Moreover, a deeper understanding of the system's environmental performance, its natural variation, and how these factors shape symbiotic function must be combined with functional studies that focus on interactions within the leaf cavity. Finally, open questions concern the broader cavity microbiome: do other bacterial residents show a consistent co‐phylogenetic signal with the cyanobionts? Are they transient bystanders – occasional passengers inherited only in particular environmental conditions – or critical, co‐inherited components of the cavity community? Addressing these questions will require fine‐grained sampling across developmental stages, the integration of diverse omics approaches, and detailed physiological analyses of both host and cyanobiont. Fortunately, the stage is set, and the tools are at hand.

## Competing interests

None declared.

## Author contributions

SdV conceived the idea of the manuscript and conceptualized the figures. Together, SdV, DB and ESL wrote the manuscript. SdV and ESL provided and assembled the photographs and micrographs of *Azolla* and its cyanobiont into a figure. All authors read and approved the manuscript.

## Disclaimer

The New Phytologist Foundation remains neutral with regard to jurisdictional claims in maps and in any institutional affiliations.
